# A case of laparoscopic appendectomy for appendiceal bleeding

**DOI:** 10.1186/s40792-023-01760-2

**Published:** 2023-10-16

**Authors:** Takuya Nakashima, Bun Sano, Aiko Ikawa, Kakeru Tawada, Tomohito Shinoda, Shinya Ohno, Reo Tachikawa

**Affiliations:** Department of Surgery, Takayama Red Cross Hospital, 3-11 Tenman-cho, Takayama-shi, Gifu, 506-8550 Japan

**Keywords:** Appendectomy, Appendiceal bleeding, Appendix diverticular bleeding

## Abstract

**Background:**

Appendiceal bleeding is very rare, accounting for about 0.4% of all lower gastrointestinal bleeding. We present a case of laparoscopic appendectomy in a patient with a diagnosis of appendiceal bleeding.

**Case presentation:**

A 71-year-old man came to our hospital with a complaint of bloody stools. He had progressive anemia and persistent fresh bloody stools, so he underwent lower gastrointestinal endoscopy. Active bleeding was confirmed from the orifice of the appendix, but the bleeding could not be stopped even with clips, so an emergency laparoscopic appendectomy was performed. His postoperative course was good, and he was discharged on the third postoperative day. Although the pathology results did not allow identification of the source of the bleeding, an appendiceal diverticulum was observed, and appendiceal diverticular bleeding was suspected.

**Conclusion:**

Appendiceal bleeding is often difficult to stop endoscopically, so appendectomy should be performed as soon as possible.

## Background

Lower gastrointestinal bleeding can be caused by colonic diverticular bleeding, ischemic enteritis, and anorectal lesions such as hemorrhoids, tumors, and inflammatory bowel disease, but the appendix is very rarely the source of bleeding. We describe a case of emergency laparoscopic appendectomy performed in a patient diagnosed as having appendiceal bleeding by lower gastrointestinal endoscopy.

## Case presentation

**Fig. 1:**
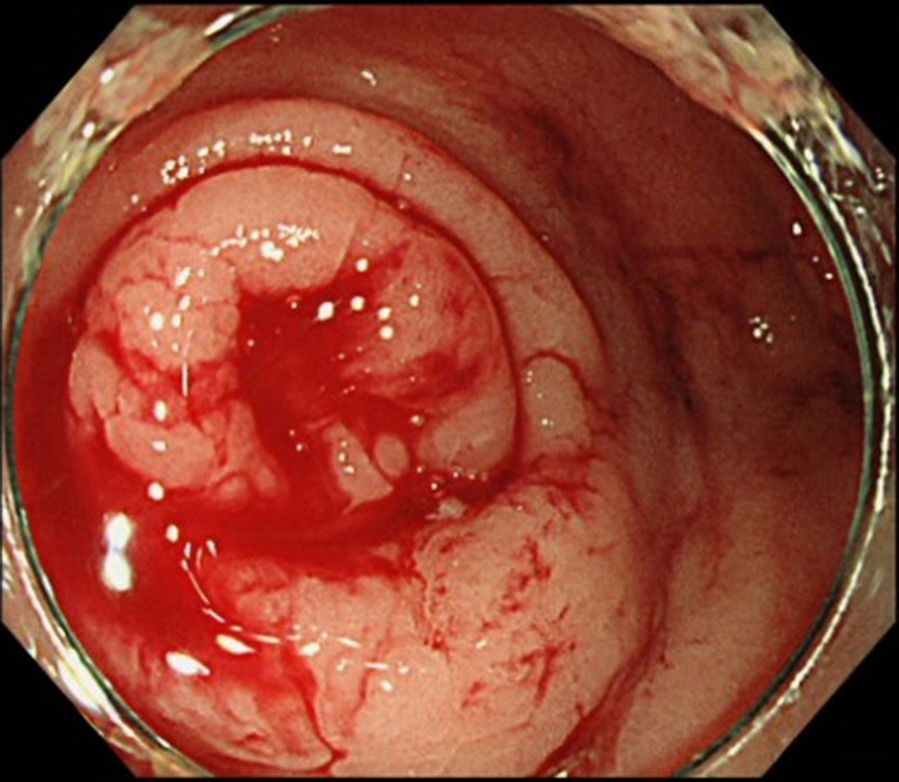
Explanation of the lower gastrointestinal findings. Bleeding is observed from the orifice of the appendix

**Fig. 2:**
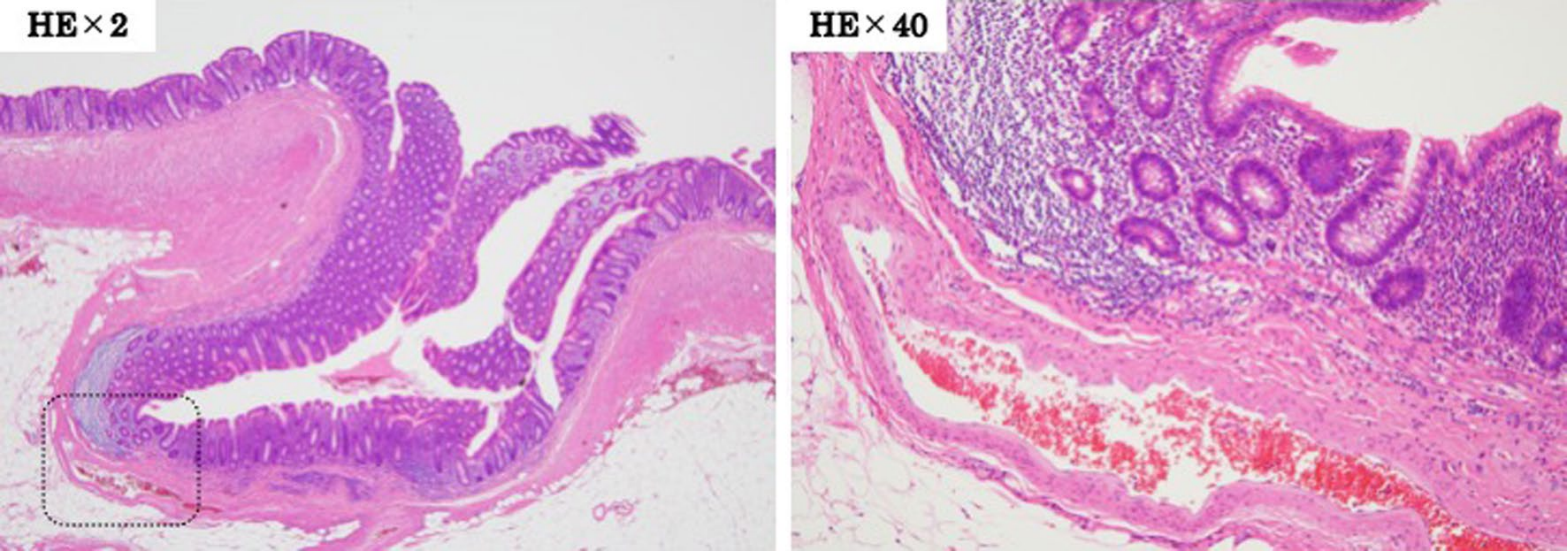
Histopathological examination findings. An appendiceal diverticulum is present, but no obvious source of bleeding can be identified. An artery was found near the appendiceal diverticulum, and it was determined to be the likely cause of the appendiceal diverticulum bleeding

A 71-year-old man with no specific medical history had constipation for 3 days, and bloody stools containing blood clots were observed one day before he visited the hospital. He continued to have bloody stools the next morning, so he visited our hospital. Abdominal findings were normal. Blood tests showed a hemoglobin of 10.5 g/dL and progressive anemia. Lower gastrointestinal endoscopy was performed because a rectal examination revealed the presence of fresh bloody stools with clots. The endoscopic examination revealed fresh blood from the anus to the cecum and active bleeding from the appendiceal orifice (Fig. 1). Hemostatic clips were used to stop the bleeding, but as hemostasis could not be achieved, we decided to perform an appendectomy. A plain CT scan performed before the endoscopic examination showed no significant findings, and the appendix was not swollen (Fig. 2).

Surgery was performed laparoscopically. The operation time was 36 min and blood loss was minimal. The appendix was normal with no enlargement. Because the clip used for hemostasis was located at the base of the appendix, an appendectomy was performed using an automatic suturing device to partially resect the cecum, taking care not to entrap the clip. The patient had a good postoperative course and was discharged on the third postoperative day.

Histopathological examination showed no inflammatory cell infiltration in the appendix. A diverticulum was observed in the appendix, but there was no vascular malformation or disruption, and the source of the bleeding could not be identified. An artery ran in close proximity to the appendiceal diverticulum, and we determined that appendiceal diverticular bleeding was the most likely cause of the appendiceal bleeding.

## Discussion

There are many causes of lower gastrointestinal bleeding, including tumors, inflammatory bowel disease, infectious bowel disease, ischemic enteritis, and other blood flow disorders, but the responsible site is very rarely the appendix. The appendix is reported to be responsible for 0.4% of all lower gastrointestinal bleeding [1]. In this case, the cause of the appendiceal bleeding was not clear from the histopathological results, but an artery ran in close proximity to the appendiceal diverticulum, suggesting that the cause was appendiceal diverticular bleeding. Appendiceal diverticulum was first described by Kelynack in 1893 [2], and Lim et al. reported that 1.74% of patients who underwent appendectomy had an appendiceal diverticulum [3]. Appendiceal diverticulum is classified into true diverticulum, which has a full-layered structure, and pseudodiverticulum, which lacks the intrinsic muscular layer. True diverticula are thought to be related to deformity due to the duplication of the appendix, a remnant of the yolk duct, or adhesions [4], but they occur congenitally and the details of their cause have not yet been clarified. Pseudodiverticula are thought to be formed by increased appendiceal luminal pressure, and pseudodiverticula account for more than 95% of all diverticula [5]. Therefore, the perforation rate is high in appendiceal diverticulitis, and complicated appendicitis often follows. A search on PubMed revealed a few cases of appendiceal hemorrhage caused by an appendiceal diverticulum [6–8], but nine cases have been reported in Japan, including the present case (Table 1). The median patient age was 62.7 years. All patients were male, which may be due in part to the fact that the male-to-female ratio of appendiceal diverticulum is 1.8:1 [3], indicating that the incidence is higher in males.

**Table 1:** Cases of appendiceal bleeding reported in Japan

No.	Author	Year	Age	Sex	Treatment	Pathological findings	References
1	Akimaru	1988	73	F	Appendectomy	Ulcer	[9]
2	Yamada	2000	70	M	Appendectomy	Appendiceal aneurysm	[10]
3	Nishi	2001	71	M	Appendectomy	Appendiceal diverticular bleeding	[11]
4	Kyokane	2001	76	F	Appendectomy	Angiodysplasia	[12]
5	Yamanaka	2002	79	M	Ileocecal resection	MALT lymphoma	[13]
6	Ueda	2004	75	F	Endoscopic hemostasis → Appendectomy	Cause unknown	[14]
7	Ueda	2004	71	M	Appendectomy	Cause unknown	[14]
8	Ogi	2006	44	M	Appendectomy	Cause unknown	[15]
9	Mori	2006	43	F	Ileocecal resection	Appendiceal endometriosis	[16]
10	Hori	2007	76	F	Ileocecal resection	Appendicitis	[17]
11	Shinozaki	2007	34	M	Appendectomy	Ulcer	[18]
12	Saida	2009	50	F	Endoscopic hemostasis → Appendectomy	Cause unknown	[19]
13	Yoshizawa	2009	0	M	Appendectomy	Appendicitis	[20]
14	Yahagi	2011	75	M	Appendectomy	Dieulafoy's lesion	[21]
15	Arai	2012	51	M	Laparoscopic appendectomy	Angiodysplasia	[22]
16	Horioka	2012	70	M	Appendectomy	Cause unknown	[23]
17	Horioka	2012	42	M	Appendectomy	Cause unknown	[23]
18	Iura	2012	40	M	Appendectomy	Appendiceal diverticular bleeding	[24]
19	Amada	2013	40's	M	Endoscopic hemostasis → Appendectomy	Cause unknown	[25]
20	Shimada	2014	59	M	Laparoscopic appendectomy	Appendiceal diverticular bleeding	[26]
21	Hokimoto	2014	28	M	Laparoscopic appendectomy	Cause unknown	[27]
22	Yoshida	2015	56	M	Appendectomy	Appendiceal diverticular bleeding	[28]
23	Morioka	2015	82	M	Endoscopic hemostasis → Appendectomy	Appendiceal diverticular bleeding	[29]
24	Hobo	2015	24	M	Laparoscopic appendectomy	Ulcer	[30]
25	Futai	2015	45	M	Endoscopic hemostasis → Appendectomy	Appendiceal laceration caused by food	[31]
26	Nakao	2015	30	M	Laparoscopic appendectomy	Ulcerative colitis	[32]
27	Hirai	2016	33	M	Laparoscopic appendectomy	Cause unknown	[33]
28	Niwano	2016	84	M	Laparoscopic appendectomy	Mucinous cystadenoma of the appendix	[34]
29	Kato	2017	20	M	Laparoscopic appendectomy	Cause unknown	[35]
30	Ogawa	2018	63	M	Laparoscopic appendectomy	Appendiceal diverticular bleeding	[36]
31	Tanaka	2020	73	M	Laparoscopic appendectomy	Appendiceal diverticular bleeding	[37]
32	Takahashi	2020	49	M	Laparoscopic appendectomy	Appendiceal diverticular bleeding	[38]
33	Maeda	2021	90	M	Laparoscopic appendectomy	Cause unknown	[39]
34	Hino	2022	40's	F	Endoscopic hemostasis → Laparoscopic appendectomy	Cause unknown	[40]
35	Fujita	2022	76	M	Laparoscopic appendectomy	Ulcer	[41]
36	Our case	2023	71	M	Laparoscopic appendectomy	Appendiceal diverticular bleeding	

MALT : mucosa-associated lymphoid tissue

We found 36 cases of appendiceal bleeding reported in Japan [9–41], including the present case. The causes were the aforementioned appendiceal diverticulum in 9 cases (25%), ulcer in 5 cases (13.9%), appendiceal tumor in 2 cases (5.6%), angiodysplasia in 2 cases (5.6%), appendicitis in 2 cases (5.6%), and dietary appendiceal tear, aneurysm, ectopic endometriosis, and ulcerative colitis in 1 case (2.7%) each. The cause of appendiceal bleeding in the remaining 12 cases was unknown. As initial treatment, 29 patients underwent surgical appendectomy, 6 patients underwent endoscopic hemostasis [14, 19, 25, 29, 31, 40], and 1 patient underwent interventional radiology to confirm hemostasis. However, 2 of the 6 patients who underwent endoscopic hemostasis required emergency surgery due to rebleeding [19, 25]. As in the present case, the presence of the hemostatic clip at the base of the appendix forced the area of resection to be expanded to resect a portion of the cecum. Even if hemostasis is achieved with a clip, appendicitis may occur due to obstruction of the appendiceal orifice. Similarly, the possibility of appendicitis is increased when hemostasis is obtained by injecting contrast media such as barium. Therefore, surgical appendectomy should be the first-line treatment for appendiceal bleeding. There is insufficient evidence regarding the use of barium filling for hemostasis of diverticular hemorrhage, and because of the risk of perforation, it is preferable not to use this technique. The Japanese Guideline for Colonic Diverticular Bleeding [42] does not recommend barium filling for the purpose of hemostasis for diverticular bleeding.

Interventional radiology is also a treatment option, but there have been reports of rebleeding [12], and even when hemostasis is achieved, it is desirable to perform appendectomy as soon as possible.

## Conclusion

We report a case of laparoscopic appendectomy for appendiceal bleeding, which should be treated with surgical resection as soon as possible.

## Data Availability

Not applicable.

## References

[1] Collins D (1963). 71,000 human appendix specimens. A final report, summarizing forty years’ study. Am J Proctol.

[2] Kelynack TN (1893). A contribution to the pathology of the vermiform appendix.

[3] Lim CSH, Cheah SY, Kwok AMF, Ravindran P, Chan DL (2020). Systematic review and meta-analysis of the association between diverticulosis of the appendix and neoplasia. ANZ J Surg.

[4] Abdullgaffar B (2009). Diverticulosis and diverticulitis of the appendix. Int J Surg Pathol.

[5] Ng JL, Wong SL, Mathew R (2018). Appendiceal diverticulosis: a harbinger of underlying primary appendiceal adenocarcinoma?. J Gastrointest Oncol.

[6] Vesa TS, Hosseini-Carroll P, Manas K (2014). Diverticular hemorrhage of the appendix. Gastroenterol Hepatol (N Y).

[7] Norman DA, Morrison EB, Meyers WM (1980). Massive gastrointestinal hemorrhage from a diverticulum of the appendix. Dig Dis Sci.

[8] Mullen JT (1979). Mucocele of the appendix associated with hematochezia. South Med J.

[9] Akimaru K, Ueda Y, Umakoshi M, Shouji T (1988). A case of massive appendiceal bleeding. Progr Acute Abdom Med.

[10] Yamada T, Fujimura M, Hirano M, Kinoshita T, Watarida S, Shirotsuka M (2000). A case of common iliac artery aneurysm causing ilioappendiceal fistula. J Jpn Surg Assoc.

[11] Nishi M, Ishizuka D, Maruta K, Kaneda S, Shimizu N, Watanabe K (2001). A study of six cases with appendicular diverticulosis. J Colon Exam.

[12] Kyokane T, Akita Y, Katayama M, Kitagawa Y, Sato Y, Shichino S (2001). Angiodysplasia of the appendix. Am J Gastroenterol.

[13] Yamanaka H, Okazima A, Sugiura T, Kitagawa Y, Kono H, Matsuura Y (2002). A case of mucosa-associated lymphoid tissue lymphoma of the appendix vermiformis. J Jpn Surg Assoc.

[14] Ueda K, Abe K, Tabata M, Katakami T, Maruta K, Yamada M (2004). Two cases of appendiceal bleeding diagnosed by colonoscopy. Prog Dig Endosc.

[15] Ogi M, Kawamura Y, Konishi F, Miyatani H, Yamada S (2006). Idiopathic hemorrhage from appendix. J Jichi Med Univ.

[16] Mori S, Kishimoto H, Tauchi K (2006). A case of appendiceal endometriosis presented with melena. J Jpn Surg Assoc.

[17] Hori K, Uchino R, Hanada N, Kusano S, Hayashida Y, Sakashita N (2007). A case of appendicitis with repeating melena. J Jpn Surg Assoc.

[18] Shinozaki H, Takahashi O, Morita Y, Takano S, Nagashima Y (2007). A case of bleeding from the appendix treated with appendectomy. J Jpn Surg Assoc.

[19] Saida Y, Nakamura Y, Enomoto T, Nakamura Y, Katagiri M, Takabayashi K (2009). A case of bleeding from the appendix treated with clipping. Prog Dig Endosc.

[20] Yoshizawa K, Yoshizawa J, Machida M, Takamizawa S, Momose Y (2009). A case of neonatal appendicitis with melena. J Jpn Soc Pediatr Surg.

[21] Yahagi M, Takahashi M, Yabuno T, Okamoto N, Kito F, Hayashi H (2011). A case of appendiceal bleeding due to Dieulafoy’s lesion. J Jpn Surg Assoc.

[22] Arai S, Oda K, Nunomura M, Ando K, Shiobara M, Sai G (2012). A case report of appendicular angiodysplasia with lower gastrointestinal hemorrhage. Jpn J Gastroenterol Surg.

[23] Horioka K, Oohata Y, Mitsuoka K, Jimi S, Kamei T (2012). Two cases of acute lower gastrointestinal bleeding from the appendix. J Jpn Surg Assoc.

[24] Iura T, Hirakawa K, Matsumoto T, Sumiyoshi K, Nakashima Y (2012). Bleeding from the diverticula of the appendix, report of a case. Stomach and Intestine.

[25] Amada E, Fujita K, Minagawa T, Ichisaka S, Sakuragawa T, Mori K (2013). A case of appendiceal bleeding with hemostasis successfully performed by endoscopic-clipping. Prog Dig Endosc.

[26] Shimada K, Sugimura Y, Kawamura H, Hatakeyama G, Nakaya T, Iijima S (2014). A case report of bleeding from appendix diverticulum. Med J Morioka Red Cross Hosp.

[27] Hokimoto N, Fujishima N, Tanida N, Oonishi K, Yamai H (2014). A case of acute appendicitis with arterial appendiceal hemorrhage. J Jpn Surg Assoc.

[28] Yoshida A, Okabe M, Noguchi J, Furukawa S, Hino H (2015). A case of simultaneously occurred bleeding from pseudodiverticulum of the ascending colon and true diverticulum of the vermiform appendix. J Jpn Surg Assoc.

[29] Morioka H, Miki A, Yoshitani S (2015). A case report of emergency surgery of bleeding from multiple diverticula in the appendix. J Jpn Soc Coloproctol.

[30] Hobo T, Omotaka S, Ogihara S, Michihata K, Isozaki M, Genki T (2015). A case of appendiceal bleeding. Prog Dig Endosc.

[31] Futai R, Mii Y, Sawa H, Oka N, Iwatani Y, Kuroda D (2015). A case of bleeding from an appendiceal laceration caused by a shrimp shell. J Clin Surg.

[32] Nakao S, Itabashi M, Bamba Y, Hirosawa T, Ogawa S, Kameoka S (2015). A case of ulcerative colitis with appendiceal hemorrhage. Tokyo Women's Med Univ J.

[33] Hirai T, Murakami M, Yamazaki K, Otsuka K, Watanabe M, Aoki T (2016). Laparoscopic appendectomy for appendiceal hemorrhage: a case report with literature review. J Jpn Soc Endosc Surg.

[34] Niwano T, Nishimura A, Iwaki T, Kawahara M, Nikkuni K (2016). A case of mucinous cystadenoma of the appendix accompanied with intestinal bleeding. Niigata Med J.

[35] Kato A, Haruki N, Chiba K, Tsumoto C, Fujita K, Denda Y (2017). A case of appendiceal bleeding. Toyota J Med.

[36] Ogawa Y, Asayama N, Nagata S (2018). Acute gastrointestinal bleeding from appendiceal diverticulitis diagnosed preoperatively by combined short-interval computed tomography and colonoscopy: a case report. Dig Endosc.

[37] Tanaka C, Yukawa T, Ikea T, Shirotsuki J, Murahashi K (2020). A case of diverticula in the appendix detected by contrast enhanced abdominal ultrasonography and colonoscopy. J Jpn Soc Coloproctol.

[38] Takahashi K, Makita F, Kurabayashi M, Yoshinari D, Kobayashi M, Tanahashi Y (2020). Laparoscopic appendectomy for appendiceal diverticular bleeding: a case report. Kitakanto Med J.

[39] Maeda U, Saito S, Ohuchi M, Tamaoki Y, Nasu J, Baba H (2021). Appendiceal bleeding in an elderly male: a case report and a review of the literature. Surg Case Rep.

[40] Hino T, Oshima Y, Sugiyama H, Sakashita F (2022). Idiopathic appendiceal hemorrhage diagnosed by active bleeding on CT and colonoscopy: a case report. Jpn J Abdom Emerg Med.

[41] Fujita H, Hirai K, Takeshima J, Ichikawa J, Ohe H, Mitsuyoshi A (2022). A case of appendiceal hemorrhage treated by laparoscopic appendectomy. J Jpn Surg Assoc.

[42] Nagata N, Ishii N, Manabe N, Tomizawa K, Urita Y, Funabiki T (2019). Guidelines for colonic diverticular bleeding and colonic diverticulitis: Japan Gastroenterological Association. Digestion.

